# Lesions of Sensations and Volition

**Published:** 1835

**Authors:** T. N. Maddox

**Affiliations:** Louisville, Kentucky


### Art. III.—Lesions of Sensation and Volition.

*History of a case of Cerebral Disease, attended with many uncommon lesions of Sensation and Volition.* By T. N. Maddox, M. D. of Louisville, Kentucky. (Communicated through Prof. Harrison.)

C. W. T. æt. 26, has rather a robust appearance, and has been very susceptible to cold and consequent coughs, since 1824, at which time she had scarlatina. On the 23rd March 1826 her father died of a paralytic stroke; she had then a "fit" which lasted five hours, and which was followed by a stupid dull sensation of body, a prickly feeling in the right arm, leg and side, and an acute pain with sense of pressure on the superior and posterior part of the head. August 20th, 1835, she was attacked by an intermittent disease of obscure character, of the tertian type; attended by pain in the head, back and knees; cold feet and legs; and a numbness and prickly sensation in the right side. When taken she was at her brother's in the country—where she remained ten days: during her stay she took two doses of calomel and one of salts, without improvement or change: she then returned to town, and took another dose of calomel without benefit. On Thursday, September 10, instead of an expected chill, she was convulsed, at 3 P. M., particularly in the upper extremities, more in the right than left side; feet motionless; face flushed; copious discharge of frothy saliva from the mouth; teeth clenched; suppression of urine during the paroxysm, and for forty-eight hours thereafter. The "fit" lasted four hours. At its termination the power of articulation was entirely gone; she complained, in writing, that half her tongue was cold, stiff and paralysed, though she protruded it and masticated her food with facility; her appearance was nearly natural, she rose when her bowels were moved, ate something, and expressed, in writing, the fear that she should never again be able to speak.

The following Saturday, at 3 P.M. she had another paroxysm, which lasted five hours and a half. The convulsions were more general and more violent, than in the preceding paroxysm, particularly in the feet; at its subsidence she complained of violent pain in the top of the head, which felt as if it were alternately opened and closed, with a stiffness and loss of power of the right shoulder. On Sunday at noon, she had another paroxysm, which continued until 8 P. M. It left her hemiplegic on the right side, except the thumb and two smaller fingers, with which she continued to write. She suffered from some pains in the bowels, which lasted several days, and was unable to rise from bed. On Tuesday, at 8 P. M., she had another paroxysm, which continued four hours, deprived her of the use of the thumb and smaller fingers of the right hand and left her deaf in the right ear. The convulsions were uncommonly violent, and she apparently ceased to breathe for twenty minutes at a time. Her attendants believed that she was dead, which error was only corrected by the continuance of the heart's pulsations. On Thursday she had another paroxysm, which left her deaf. From this period, she had a severe pain in the eyes, which greatly augmented the lachrymal secretion — the tears were occasionally so abundant, that they coursed their way over her cheeks. During the week she had several paroxysms which were so mild that they made no distinct impression on the minds of her attendants, neither did they aggravate her sufferings. On Thursday, September 24th, she complained of giddiness; was somewhat delirious in the morning; made in writing incoherent remarks; tore her hair; kicked violently with her left foot; threw off the bed clothes, and induced the family to conclude that she was insane. At 10 A. M. she had a violent paroxysm, which lasted until 5 P. M. — during this period she stared wildly; her head drawn backwards; opened and closed her eyes with great difficulty, which remained in either condition for hours at a time. Light seemingly did not affect them, nor did the contiguity of foreign bodies cause her to wink them. During this paroxysm a very copious and inspissated discharge of tears (in consistence resembling pus) took place, which is described as possessing a scalding property. This discharge continued eight days. At the subsidence of this paroxysm she was blind, though her eye-lids were as widely separated as possible. Her memory was then more acute than in perfect health; her sensibility to impressions extraordinarily delicate; and the sense of touch in the left hand, and of feeling in the left arm and side of the face astonishingly discriminative: for instance, she readily recognised all persons with whom she had previously had an interview, by some peculiarity of conformation or dress; and distinguished physicians from other persons by their manner of examining as to the dryness or moisture of the skin. After filling a slate with writing, she traced line after line, expunging superfluous words, and correcting mistakes of every description, with as much accuracy as she could have done with her eyes. The right extremities were wholly immovable. On the following Monday she was, bled by her physician, with the consent of others, to three ounces, which removed the prickly sensation of the right side. A day or two after the last paroxysm, she commenced spitting blood, which continued in greater or less abundance until the 1st October, when she had a very severe paroxysm; commencing at 4 P. M., and lasting two and a half hours. It was exceedingly violent, and attended by extreme difficulty of breathing and swallowing. She was frequently and violently convulsed, more in the left than right side; discharged frothy saliva from the mouth in abundance; with clenched teeth and a fixed immobility of the right extremities. It was apparently produced by grief, at the recognition of a fond mother; her eye-lids have been closed since this paroxysm, and are not influenced by the volitions of the patient. She thinks that at the subsidence of this paroxysm, she had a blood-vessel of the breast ruptured, from the fact that she spit quantities of blood; the quantity was so great that she was constantly engaged, either in spitting or swallowing, and was accompanied by an apprehension of immediate suffocation, of four or five days duration, during which time she required the room, to be freely ventilated and fanning from her attendants, whom she besought not to place her in a recumbent position, as she was confident that in that position suffocation would inevitably ensue. She was unconscious during each of the paroxysms. Her physician repeatedly announced to the family that the case would have a fatal termination, and for the two last weeks had discontinued his visits and prescriptions: — we learned these particulars from the family at our first visit. On Monday, October 26th, Dr. Murray was requested to visit this lady, and was so kind as to invite me to see the case with him, which we saw for the first time on Tuesday, October 27th. She solicited us to take charge of her, stating that her former physician had deserted her. Perhaps it will not be improper to state here, that four or five physicians, who visited her previous to this period, believed that the case was irremediable, and openly announced the opinion. We believed that death would probably close the scene, and asked permission to examine the head in that event.

Oct. 27. She is as for several weeks past, dumb and blind; is propped up in bed; countenance nearly natural; eyes closed; tremors of the eye-lids; an inability to separate the eye-lids, or direct the pupil downward when separated; bowels torpid; pulse seventy-five, laboring; head hot; feet cold; skin preternaturally warm and moist; tongue nearly natural; urinary discharge tolerably free and unusually limpid; sleeps little — perhaps an hour each night, and is much disturbed by unpleasant dreams; though absent and forgetful when well, her memory is now exceedingly acute, and her expressions unusually felicitous; no appetite, though she takes food and stimulants to remove an unpleasant nausea, and support her exhausted energies; her friends communicate to her by writing on the left hand or face; writing on the face, however, produces uneasiness. She frequently anticipates the meaning before the communication is finished, and eagerly seizes the pencil to reply. She writes very correctly, and perfectly straight, with her left hand, and rectifies any error of orthography or punctuation with facility. Her hand is so tremulous, that wine and water or coffee, must be freely used to enable her to write a communication. She understands nothing written on the right hand; she suffers from pain in the head, which is often acute; and from pain in the back, which is aggravated by moving or being moved. By the sense of touch, she readily recognises her friends, and all persons she has seen the second time; and when visited by a particular friend, burst into tears. She has regularly menstruated at intervals of three weeks, since the establishment of this function, and has much pain and restlessness on these occasions. It continues usually a week. The week preceding its appearance she is languid, indisposed to action, and frequently vomits, more especially on the day previous to its accession. It leaves her weak, exhausted and depressed, which state lasts usually another week, so that she is scarcely ever well.

This individual has generally a keen appetite in health, which she has been in the habit of indulging; possessed a robust and vigorous constitution, led a sedentary life, and usually enjoyed excellent health. She is constitutionally gloomy and despondent, and, like all persons of this temperament, is subject to occasional exaltations and depressions of spirits. In her gloomy moods she has often withdrawn from the society of friends, and brooded with intense fixedness on her multiplied calamities. In the morning of life, a kind and indulgent father provided for her; but she was scarcely able to appreciate his kindness, ere death snatched him from her. The distribution of property, according to will, was unsatisfactory to some of the heirs, who believing that the law would dispose of it more equitably, endeavored to nullify the will. This attempt bred family dissensions, and ended in disappointment. She then sought an asylum from family contentions in the house of a favorite brother, who kindly received, and carefully educated her — her education was no sooner completed, than death tore from her this brother. Calamity thus followed calamity, until all her fondest hopes were blasted — all her brightest anticipations disappointed, and she left an unfriended being.

Her grief, her seclusion, her sedentary habits, her destitution of friends, the cruelty of a brother, and the faithlessness of a connection, made her an easy prey to melancholy; aggravating her original gloominess, and rendering her more than ever the victim of despondency. In addition to this, she frequently suffered from the most excruciating head-aches — an effect to the production of which the preceding causes are fully adequate, and which is always aggravated, by a simultaneousness in the occurrence of these circumstances. Her indispositions are all felt most sensibly in the head, and this was particularly the case in her late intermittent attack. Had she been properly treated, the convulsions, perhaps, would not have occurred.

We conceived that the convulsions, and subsequent phenomena, were the result of a lesion of circulation of the encephalic vessels; which, but for the copious discharge of blood from the lungs, would, in all probability, have resulted in fatal Apoplexy. With this view we directed that her head should be shaved, and that three cups be applied to its occipital portion; her feet to be rubbed with a saturated decoction of cayenne, and a pill containing two grains of aloes, two of scammony, and one of gamboge, at bed time, and, in case it failed to operate by morning, two more.

28th. The three pills procured two evacuations, of good character, after cupping less pain and tension of the head, particularly the eyes; skin cooler. ℞. Cloths wrung out of cold water to head; external warmth to feet; and two more pills at bed time.

29. During the night, the medicines operated twice; slept better than usual; less pain and heat of head; less languor; capable of greater effort; "feels sensibly better;" pulse seventy-five, less labored, more compressible, less contracted; eyes painful; less tremor of the eye-lids; skin cooler; ℞. three cups to right side of head; a pill at an hour's interval, until she has taken three; cloths wrung out of iced-water to head; continue stimulating applications and external warmth to feet; diet light, unirritating and digestible: — 6 P. M., cupping has lessened pain in the head:—℞. Sulph. Morph. gr. ¼, Scammon. grs. ij., Aloes grs. ij., ft. pil. i. at bed-time.

30. Slept better last night, and was less disturbed by dreams; had two alvine dejections, of a dark colour; pulse freer and more compressible; tongue clean; asked for tea and corn cakes, so soon as she awoke this morning; has more control over the muscles of the right extremities; some pain and soreness about the right elbow, more especially on moving the arm; "imagines that she sees a little, though she believes it is all imagination;" is delighted that she can partially elevate the eye-lids; has still some pain and pressure in the occipital region. ℞. three cups to posterior portion of the head; three pills at bed-time; friction of right elbow and leg with decoction of cayenne; continue cold applications to head, and external warmth to feet.

31. "Some soreness of the head this morning;" no pain; much pain in back and bowels; medicine operated several times—once very copiously; pulse eighty-two, freer and more compressible; says "that she is much better;" "that she has had a cerebral affection for eight years; that she desired her head shaved and cold applications to it when first taken, which were denied her; regrets that she has been so long deprived of the facilities of seeing, hearing and speaking—but hopes soon to furnish her friends and physicians with an account of the origin, progress and treatment of her case." ℞. three cups to head; ℞. Aloes Socot., Scammon. aa grs. ij., Gamboge gr. j., Sulph. Morph. gr. ¼, in a pill at bed-time; a blister to each ankle. ℞. Tinct. Opii, Aqua Ammon., Ol. Olivar., aa ℥j., Ol. Caryoph. ʒjss., ft. Liniment, for right arm and leg.

Nov. 1. Patient slept more and better last night, has more control over the muscles of the right arm and leg; can elevate the eye-lids considerably; of which there is now no tremor, directing the pupils downwards at the same time, so much as to make it partly visible; "says that the air or light gives her pain in the eyes;" all odors are disagreeable; appetite improving; skin pleasantly cool; "is annoyed by the walking of persons across the floor, which jars her bed;" bowels not moved to-day; pain in the lumbar region. ℞. three pills this morning, and another at bed-time, with a grain of the Extr. Hyoscyam.—continue liniment, cold applications to head, and external warmth to feet. We directed that the room should be darkened, and the number of visitors lessened.

2. Slept well; medicine operated four times; she "feels extremely weak;" pulse eighty, small, and gives rather a jerking sensation; no pain, except in the lumbar region, which is not very annoying; no urinary discharge to-day. She affirmed, that a medical gentleman, who visited her with us was a physician, though she never saw or heard of him before, is "exceedingly anxious to see and speak again;" "expects to receive much of the world's censure;" wept when a friend requested her to correct a mistake in her communication, "because she thought it equivalent to a suspicion of her candor, and an improper indulgence of curiosity." ℞. a pill at bed-time, with ¼ gr. Sulph. Morph., three cups to side of head; continue cold applications, external warmth and liniment; diet, milk and mush, tea and thin cakes, &c.

3. Patient roused the family at day-dawn, "to tell them she could see, though unable to distinguish objects;" pupils much dilated; the free admission of light causes no contraction of the iris, but produces pain; feet warmer; some giddiness of the head without pain; has the usual premonitions of menstruation; discharged a quantity of high-coloured urine this morning; increasing control over the right arm and leg:—after cupping, "said her tongue was very sore on the right side, but not so stiff; and that she had a disagreeable taste in her mouth she could not describe." In her ecstacy at seeing, she forgot her breakfast, which she asked for so soon as she had seen her friends and physicians. ℞. Gum. Aloet, Gum Myrrh. Sulph. Ferri, aa ʒss. Extr. Gentian., Pulv. Zingiber., aa ʒj., ft. pill. No. xxx,—one, morning and evening; two purgative pills at bed time; three cups to head; a blister to right thigh near the knee; continue liniment; omit cold applications and external warmth.

4. The blister was so painful that she slept but little last night; some giddiness; three alvine dejections during afternoon and night; "feels extremely weak;" pulse ninety; raised her right hand to her head without assistance; vision improving, though the effort of looking gives pain; called Rachel — an attendant, in an audible tone; can articulate but few words, mostly monosyllables; skin uniformly cool and pleasant; continue emmenagogue pills and liniment.

5. "Dr., for the first time, since I have been sick, I feel something like myself; I can see any object near to me, but it is painful; my tongue and throat on the right side are very sore, — but great is the change in my feelings for the better; I have no longer that strange feeling in my head; — if my throat were less sore on the right side, I think I could speak; something seems constantly drawing my tongue on the right side." Has more control over her arm than leg — can raise her arm to her head and change its position with facility; is fatigued from lying so long in bed, — and is anxious to sit up; bowels moved twice this morning; menstruation commenced; appetite much better; pulse eighty, softer; slept well; discerns objects to-day; the free admission of light produces pain; has great difficulty in understanding what is written on her hand, from a diminution of sensibility in it. In the afternoon complained of giddiness and appeared overcome by fatigue; continue emmenagogue medicine and liniment.

6. Had a tranquil night's rest undisturbed by dreams, menstruation ceased; less soreness of the tongue, though the effort of speaking is painful; the pupil *now* contracts when light is admitted; sees so well that she reads communications on the slate, — understands with great difficulty what is written on her hand, of which the palm and inside of the fingers are very sore; looking still produces pain; pulse softer; appetite good; continue pills as before; two cups to back part of head; liniment to back, spine, and right extremities; inhalation of the vapour of the decoction of Cayenne.

7. Last afternoon and night so much drowsiness and torpor of the brain, that she hardly remembered what she wished to say; "feels stronger;" complains of a pain on moving between right mamma and ilium; taste vitiated, sometimes bitter, sometimes salt; pulse eighty, good; writes to-day (for the first time since the fourth paroxysm) with the right hand; understands no longer what is written on her left hand; receives communications on her slate, which she reads without pain; is in fine spirits; appetite so keen that she asks for bacon;—continue pills—liniment—and vapourous inhalation: three cups to posterior portion of head.

8. We found our patient sitting up; sees well; saltish taste in the mouth; bowels once moved; tingling in the ears; sleep disturbed last night by unpleasant dreams; called Rachel, and asked for water audibly and distinctly; "thinks that she may recover;" continue pills, vaporous inhalation and liniment.

9. Less tingling — a dull roaring in the ears occasionally; bowels moved twice; soreness on the right half of the body; a desire to change bed; an indefinable susceptibility to impressions--amounting almost to peevishness: continue pills and liniment; omit vapourous inhalation: apply three cups to occipital region of the head.

10. Better.

11. Appetite voracious; spirits fine; some improvement in speaking, though her articulation is yet very imperfect; omit pills; continue liniment; three of the purgative pills at bed time:—we allowed a small piece of bacon for dinner to-day.

12. Some pain when moved; little or no soreness of the tongue or throat; speaks much more easily,—and with a little assistance walked across the room; no medicine to-day; continue liniment.

13. Thinks she heard the town bell this morning; has a constant roaring in the ears—and much hesitancy and catching in speaking, though she no longer uses the slate; hopes high; some soreness of the right thigh, which is painful when moved. ℞. three purgative pills at bed time; continue liniment.

14. Hears some words, though there are occasional intervals of indistinctness and sometimes confusion of sounds; thigh less sore and painful; medicine operated freely several times; less impediment in speaking to-day; no medicine; continue liniment.

15. Hears to day perfectly well at intervals, which is followed by periods of entire deafness of four or five minutes duration; less hesitancy in speech; is rapidly improving; says "she feels better than she has felt for ten years;" leg still painful when moved; continue liniment.

16. Better.

17. Hears and speaks almost as well as ever; leg improving; bowels in good condition; continue liniment. A physician, whom she knew for the first time during her blindness, and who was always recognised at subsequent visits, called to see her this morning with us—to our astonishment she knew him not:—her touch was no longer sufficiently delicate to distinguish him from others.

18. Hears, sees, and speaks well; right extremities getting stronger; no pain to-day.

Dec. 12. This lady continued to improve, now walks out and is apparently in good health.

Louisville, Ky., Dec. 15, 1835.

